# Integrative Network Analysis Reveals a MicroRNA-Based Signature for Prognosis Prediction of Epithelial Ovarian Cancer

**DOI:** 10.1155/2019/1056431

**Published:** 2019-06-04

**Authors:** Li Li, Haiyan Gu, Lingying Chen, Ping Zhu, Li Zhao, Yuzhuo Wang, Xiang Zhao, Xingguo Zhang, Yonghu Zhang, Peng Shu

**Affiliations:** ^1^Department of Gynecology, Beilun People's Hospital, Ningbo 315800, China; ^2^Department of Gynecology, Cangzhou People's Hospital, Cangzhou 061000, China; ^3^Department of Urology, Hefei BOE Hospital, Hefei 230041, China; ^4^Prenatal Diagnostic Laboratory, Cangzhou People's Hospital, Cangzhou 061000, China; ^5^Department of Hepatobiliary Surgery, Nanjing Jiangbei People's Hospital Affiliated to Nantong University, Nanjing 210048, China; ^6^Department of Clinical laboratory, Beilun People's Hospital, Ningbo 315800, China; ^7^Department of Infectious Diseases, Beilun People's Hospital, Ningbo 315800, China

## Abstract

**Background:**

Epithelial ovarian cancer (EOC) is a heterogeneous disease, which has been recently classified into four molecular subtypes, of which the mesenchymal subtype exhibited the worst prognosis. We aimed to identify a microRNA- (miRNA-) based signature by incorporating the molecular modalities involved in the mesenchymal subtype for risk stratification, which would allow the identification of patients who might benefit from more rigorous treatments.

**Method:**

We characterized the regulatory mechanisms underlying the mesenchymal subtype using network analyses integrating gene and miRNA expression profiles from The Cancer Genome Atlas (TCGA) cohort to identify a miRNA signature for prognosis prediction.

**Results:**

We identified four miRNAs as the master regulators of the mesenchymal subtype and developed a risk score model. The 4-miRNA signature significantly predicted overall survival (OS) and progression-free survival (PFS) in discovery (*p*=0.004 and* p*=0.04) and two independent public datasets (GSE73582: OS, HR: 2.26 (1.26-4.05),* p*=0.005, PFS, HR: 2.03 (1.34-3.09),* p*<0.001; GSE25204: OS, HR: 3.07 (1.73-5.46),* p*<0.001, PFS, HR: 2.59 (1.72-3.88),* p*<0.001). Moreover, in multivariate analyses, the miRNA signature maintained as an independent prognostic predictor and achieved superior efficiency compared to the currently used clinical factors.

**Conclusions:**

In conclusion, our network analysis identified a 4-miRNA signature which has prognostic value superior to currently reported clinical covariates. This signature warrants further testing and validation for use in clinical practice.

## 1. Introduction

Epithelial ovarian cancer (EOC) is the most lethal gynecological malignancy, characterized by molecular and pathological heterogeneity. The main pathological type is the high-grade serous ovarian cancer (HGSOC) [1]. Despite new screening and treatment strategies, the prognosis for EOC remains poor. The main reason for the poor prognosis is late-stage presentation during diagnosis, widespread dissemination, and high recurrence rate [2]. Standard treatment for EOC is aggressive resection of the primary tumor followed by adjuvant chemotherapy. Owing to the diffuse nature of EOC, the relapse rate is high even in those who initially had a complete pathological response, and most of the patients developed chemoresistance [3]. Overall survival (OS) has improved moderately over the past 30 years, with a 5-year survival rate of approximately 30% [4].

Several studies have attempted to develop molecular signatures based on gene expression to predict EOC prognosis. However, only few molecular prognostic signatures have been developed [5–7], of which not many have been externally verified, and none of them could be directly applied in clinical practice. One of the reasons for the low prognostic efficacy is the heterogeneity of EOC during initial diagnosis. Recently, Tothill et al. [8] identified 4 subtypes of ovarian cancer with distinct molecular and clinical characteristics by unsupervised classification of the gene expression patterns and revealed that the mesenchymal subtype had the worst OS. Subsequently, the mesenchymal subtype was recapitulated in several other studies [9, 10]. Therefore, exploring the underlying determinants of the poor prognosis mesenchymal subtype could be potentially used for risk stratification and for developing more precise, targeted treatment strategies for EOC patients.

To find a better risk prediction method for EOC patients, we decided to focus on miRNAs-a class of short sequences of noncoding RNA [11], since they act as the master regulators of gene expression [12]. In this study, we applied network analysis to reveal the regulatory mechanisms underlying the mesenchymal subtype, integrating gene, and miRNA expression profiles. The master regulator analysis showed that the mesenchymal subtype was regulated by four miRNAs.

Although studies on miRNA signatures in EOC have been reported [13, 14], no research has been done by integrating the characteristics of molecular subtypes for risk stratification. We aimed to identify a miRNA-based signature by incorporating a variety of molecular modalities involved in the mesenchymal subtype for risk stratification that would allow the identification of EOC patients who might benefit from more rigorous treatments.

## 2. Materials and Methods

### 2.1. Patient Series

In this retrospective study, we performed a comprehensive analysis of 725 patients using three independent miRNA cohorts from women with EOC (Table 1). These cohorts included patients from the TCGA dataset (training cohort, N=462) [9], the GSE73582 cohort (validation set, N=133) [15], and the GSE25204 cohort (validation set, N=130) [16]. mRNA profiles were derived from the TCGA dataset consisting of 462 EOC patients [9]. The expression profiles of miRNAs and mRNAs together with the corresponding clinicopathological parameters were downloaded from Gene Expression Omnibus (GEO http://www.ncbi.nlm.nih.gov/geo/) and TCGA data portal (https://tcga-data.nci.nih.gov/tcga/). The study was performed in accordance with the Declaration of Helsinki and approved by the institutional review board of Beilun People's Hospital, Ningbo, China.

### 2.2. Expression Data Preprocessing

Microarray data of the two validation cohorts, normalized using the robust multiarray analysis (RMA), were downloaded from GEO using R package “GEOquery” (version 1.0.7) [17]. We further removed the nonbiological batch effects of the GSE25204 cohort using ComBat function in R package “sva”. The Cancer Genome Atlas (TCGA) dataset, including 462 matched miRNA and mRNA expression profiles, was downloaded from the TCGA data portal by “TCGAbiolinks” R package [18] and normalized using voom function in* limma* R package [19]. For each dataset, the expression profiles were transformed from probe sets or entrez sets to gene symbols.

### 2.3. Regulatory Network Inference

We have applied regulatory network inference to study the regulatory mechanisms of the mesenchymal subtype by integrative analysis of miRNAs and mRNA expression profiles. Together, we analyzed 462 patient samples with both miRNA and mRNA expression profiling data in the TCGA cohort. Thirty-six miRNAs (|log2 fold change| > 0.5, Benjamini-Hochberg adjusted* p* < 0.05) and 1659 genes (|log2 fold change| > 0.25, Benjamini-Hochberg adjusted* p* < 0.05) were differentially expressed in the mesenchymal subtype compared with the other three subtypes. The miRNA and mRNA expression profiles were normalized independently and subsequently integrated for network inference in the R “RTN” package [20]. We performed master regulator analysis (MRA) [21] to test epithelial–mesenchymal transition (EMT) genes significantly overrepresented in each miRNA's regulon. Four microRNAs of top significance (Benjamini-Hochberg-adjusted* p* < 0.05) were selected as the master regulators of the mesenchymal subtype.

### 2.4. miRNA Signature Construction and Risk Score Calculation

Four miRNAs were differentially expressed in the poor prognosis subtype and were identified as the powerful determinants of the mesenchymal-specific gene expression, including EMT-related genes. Based on these four miRNAs, a cox-model was established as follows: risk score = (0.0685×miR-449a) + (0.1258×miR-409-3p) + (-0.0081×miR-200a) + (-0.1176×miR-508-3p).

### 2.5. Statistical Analysis

Wilcoxon signed-rank test was used to identify differentially expressed miRNAs between different groups. Patients were dichotomized into high and low-risk groups by the median value of risk scores. The Kaplan-Meier curves were plotted to compare the survival analysis by using a log-rank test. Additionally, univariate and multivariate cox proportional hazard regression models were used to calculate hazard ratios to evaluate the prognostic significance of clinicopathological variables and the 4-microRNA signature. Statistical significance was denoted by *∗p* < 0.05, *∗∗p* < 0.01, and *∗∗∗p* < 0.001, and a p value of less than 0.05 was considered significant. All statistical analyses were performed using R (versions 3.4.0, https://cran.r-project.org/).

## 3. Results

### 3.1. Integrative Analysis Identifies Master miRNA Regulatory Network for the Mesenchymal Subtype

**Figure 1:**
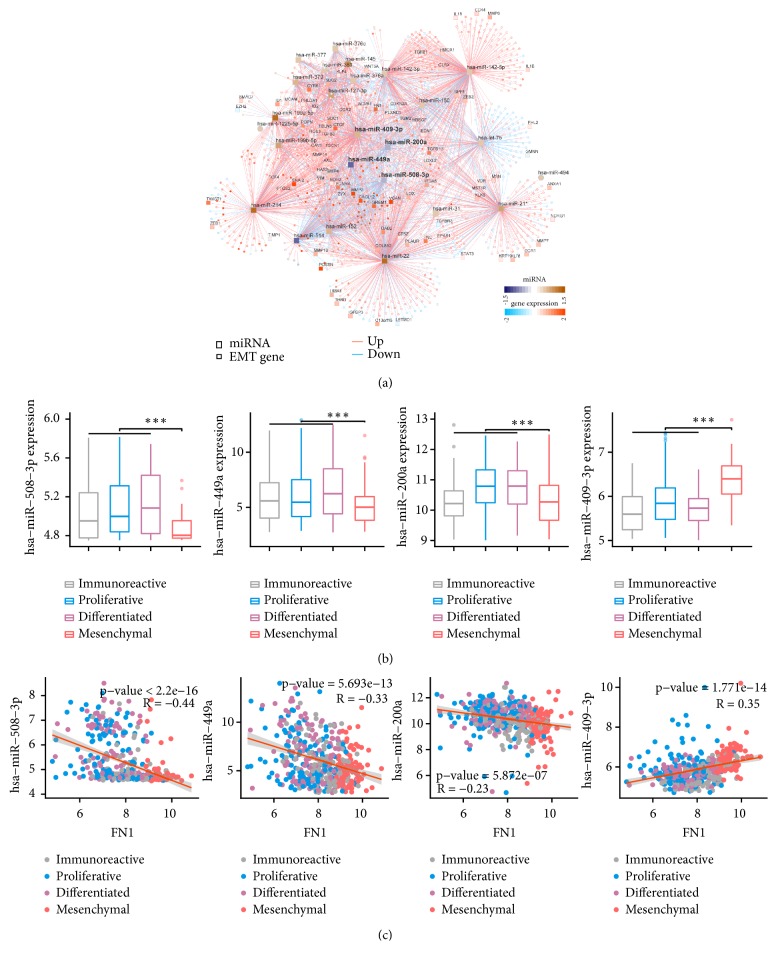
*Network inference analysis reveals four major regulatory networks of the mesenchymal subtype.* (a) The mRNA-miRNA network shows the relationships between four key miRNAs and the EMT signature genes. (b) The four-miRNA signature was significantly lower in the mesenchymal subtype in the TCGA dataset than the other three subtypes. (c) Significant correlation between FN1 expression and the four-miRNA expression in the TCGA dataset.

**Figure 2:**
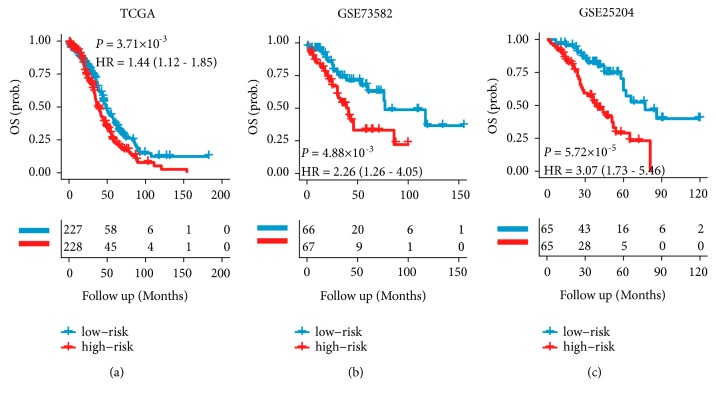
*OS stratified by risk according to the 4-miRNA signature.* The Kaplan-Meier plots show OS in patients stratified by the 4-miRNAs signature in the TCGA training cohort (a), GSE73582 validation set (b), and GSE25204 validation set (c). p values are based on log-rank tests.

EOC is a heterogeneous disease, which has been classified into four molecular subtypes with distinct molecular and clinical characteristics, of which the mesenchymal subtype was found to exhibit the worst prognosis [8–10]. To investigate the regulatory mechanisms underlying the mesenchymal subtype of EOC, we generated regulatory networks by integrative analysis [20] of miRNA and mRNA expression profiles in the TCGA cohort (Figure S1). The miRNA networks consist of miRNAs that are differentially expressed in the mesenchymal group compared with the other three subtypes (Figure 1(a)) and were found to regulate the expression of most of the mesenchymal-specific genes. Master regulator analysis (MRA) revealed four microRNAs (miR-449a, miR-409-3p, miR-200a, and miR-508-3p) as the dominant regulators in the mesenchymal subtype (Table S1), whose expression levels differ significantly between the mesenchymal and other three subtypes (Figure 2(b)). As reported previously, overexpression of the miR-200a [22], miR-449a [23], and miR-508-3p [24] is associated with the inhibition of the EMT program, whereas the high expression of the miR-409-3p [25] promotes tumor growth and the EMT program. EMT signature genes are significantly correlated with the four miRNAs, revealing that the mesenchymal property is indeed regulated by these four miRNAs (Figure 1(c) and Figures S2-3). Therefore, these four miRNAs are major regulators of the mesenchymal phenotype and can be potentially used for risk assessment of EOC tumors.

### 3.2. Development of a 4-miRNA Signature in EOC Patients

**Figure 3:**
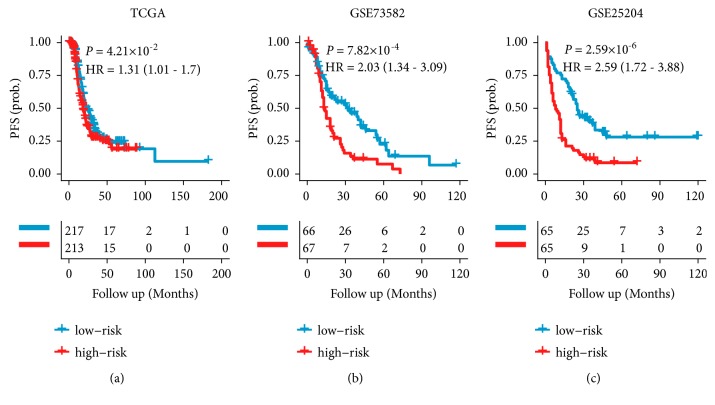
*PFS stratified by risk according to the 4-miRNA signature.* The Kaplan-Meier plots show PFS in patients stratified by the 4-miRNAs signature in the TCGA training cohort (a), GSE73582 validation set (b), and GSE25204 validation set (c). p values are based on log-rank tests.

Using the TCGA cohort, we constructed a cox-model based on the expression of these four miRNAs: Risk Score = (0.0685 × expression value of miR-449a) + (0.1258 × expression value of miR-409-3p) + (-0.0081 × expression value of miR-200a) + (-0.1176 × expression value of miR-508-3p). Of the four miRNAs, 3 were associated with improved prognosis (miR-200a, miR-449a and miR-508-3p) and one with worse prognosis (miR-409-3p). Using the 4-miRNA signature, risk scores were calculated in the TCGA cohort. Patients were significantly divided into high- and low-risk groups (median risk score as cut-off value; Table S2) in terms of OS (HR 1.44, 95% CI 1.12–1.85;* p*=0.0037; Figure 2(a)) and PFS (HR 1.31, 95% CI 1.01–1.70;* p*=0.04; Figure 3(a)). High-risk group patients showed a shorter median survival than did the low-risk group (29 months versus 31 months).

### 3.3. Validation of the 4-miRNA Signature

To confirm the prognostic power of the 4-miRNA signature, risk scores were calculated, and patients were stratified into two risk groups (Table S2) in two publicly available validation cohorts. In GSE73582 and GSE25204, patients were significantly divided into high- and low-risk groups in terms of OS (GSE73582: HR 2.26, 95% CI 1.26–4.05,* p*=0·005, Figure 2(b); GSE25204: HR 3.07, 95% CI 1.73–5.46,* p*<0.001, Figure 2(c)) and PFS (GSE73582: HR 2.03, 95% CI 1.34–3.09,* p*<0.001, Figure 3(b); GSE25204: HR 2.59, 95% CI 1.72–3.88,* p*<0.001, Figure 3(c)). In the univariate analysis, advanced stages and debulking status were significantly associated with prognosis; however, the 4-miRNA signature outperformed these clinical factors (Table 2). In GSE73582, the HR was 2.04 (95% CI 1.34–3.10,* p*=0·0008) and, in GSE25204, HR was 2.59 (95% CI 1.72–3.88,* p*<0.0001). Moreover, multivariate analysis demonstrated the 4-miRNA signature as the strongest predictor in two validation cohorts after adjusting for other clinical factors (Table 2)

### 3.4. An Association between the 4-miRNA Signature and Chemotherapy Response

**Figure 4:**
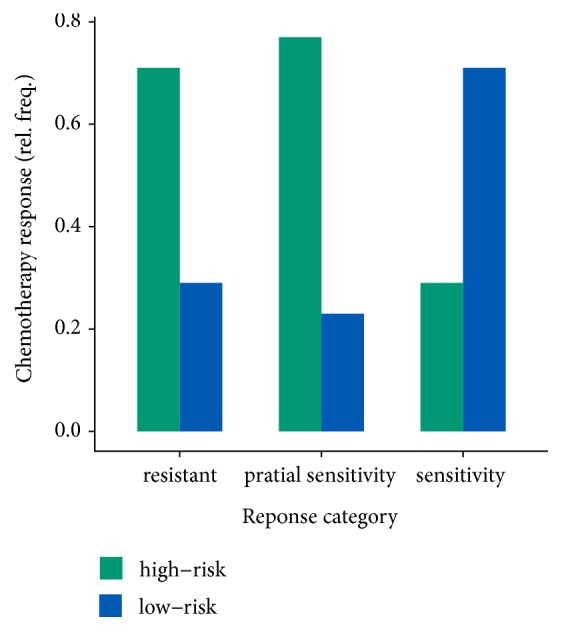
The association between the 4-miRNA signature and chemotherapy response.

To examine the association of the 4-miRNA signature with response to first-line platinum-based therapy [26], we analyzed the chemotherapy response within different risk groups in the GSE25204 cohort and identified an association between the 4-miRNA signature and chemotherapy response (*p*<0.001, Fisher's exact test; Figure 4). Low-risk group patients exhibited a high likelihood of platinum sensitivity and those patients in high-risk group tended to have a high likelihood of platinum resistance or partial platinum sensitivity. In the high-risk group and low-risk group, 30.7% and 75.3% patients, respectively, achieved platinum sensitivity.

## 4. Discussion

EOC is the leading cause of gynecological cancer deaths. Currently, clinical features such as tumor grade, histopathological classification, debulking status, and CA-125 levels are the most common criteria to evaluate the risk of HGSOC patients [27]. Although a lot of multigene prognostic signatures [9, 28–30] have been developed, the accuracy of their prognostic prediction remains uncertain. A method to identify EOC patients with a worse prognosis is urgently needed to improve the design of customized therapies.

Unsupervised classification of EOC transcriptome profiling revealed four molecular subtypes with distinct molecular and clinical characteristics [8–10]. The subtype-specific molecular portraits, especially the worst prognosis subtype-specific prognostic signature, could be potentially used for risk stratification [24, 31]. We aimed to build a widely useful signature that integrates the molecular differences seen in the poor prognosis subtype of EOC. miRNAs are short noncoding RNAs that regulate gene expression and have been demonstrated as prognostic biomarkers in EOC [32]. The development of the miRNA-based signature is based on network analysis to identify a variety of molecular modalities involved in the mesenchymal phenotype. Our 4-miRNA signature revealed the regulatory mechanisms of the mesenchymal subtype and was able to identify groups of patients with significantly poor OS and PFS. The high-risk group patients had a worse prognosis and exhibited poor response to chemotherapy, suggesting that more aggressive treatments would benefit them. The 4-miRNA signature maintained its independent prognostic power in multivariate analysis after adjusting for tumor stage and debulking status, which are established clinical factors for prognostic estimation of EOC patients.

All the four miRNAs have already been reported as having fine-tuning roles in EMT processes. Of the four miRNAs, three contribute to a favorable prognosis and one contributes to worse prognosis. Zhao and colleagues identified that miR-508-3p was involved in the regulatory network related to the EMT program in the mesenchymal subtype of EOC. Higher expression of miR-508-3p was associated with significantly better prognosis [24]. Chen et al. demonstrated that overexpression of miR-449a inhibited tumor metastasis and was associated with better disease-free survival of patients with hepatocellular carcinoma (HCC) [23], suggesting its oncosuppressive role. Lu et al. have reported that loss of miR-200a expression is associated with the EMT phenotype and promoted cell migration and invasion in pancreatic cancer [33]. However, elevated expression of miR-409-3p was observed in metastatic prostate cancer and was correlated with poor PFS [25]. The main prognostic effect of the 4-miRNA signature was associated with the regulation of the EMT program, which initiates tumor spread and progression of EOC.

Three independent cohorts with mature follow-up information were used to construct a prognostic predictor for EOC. Since we focused on the analysis of miRNAs shared by all the platforms, we may have missed other meaningful miRNAs. Before the 4-miRNA signature can be developed for routine clinical use, its limitations should be considered. First, the prognostic signature is based on expression profiles produced by microarray platforms, which are difficult to popularize for routine clinical use due to their high price, long conversion cycle, and requirement of bioinformatics expertise. Second, more datasets with full clinical annotations need to be included in the analysis for broader validation.

## 5. Conclusions

In conclusion, our network analysis identified a 4-miRNA signature which has prognostic value superior to currently reported clinical covariates. Our study represents the first attempt to integrate tumor heterogeneity and develop a risk model which could be validated in silico.

## Figures and Tables

**Table 1:** Patient characteristics.

	Training cohort (TCGA, *n*=462)	Validation cohort (GSE73582, *n*=133)	Validation cohort (GSE25204, *n*=130)
Age (years)	60 (30-87)	56 (27-82)	54 (25-85)
Stage			
I		16 (12%)	
II	21 (5%)	9 (7%)	
III	361 (78%)	105 (79%)	107 (82%)
IV	76 (16%)	3 (2%)	23 (18%)
Unknown	4 (1%)		
Grade			
1		5 (4%)	1 (1%)
2	55 (12%)	29 (22%)	25 (19%)
3	394 (85%)	82 (62%)	95 (73%)
Unknown	14 (3%)	17 (13%)	2 (2%)
Debulking			
optimal	302 (65%)	55 (41%)	21 (16%)
suboptimal	112 (24%)	77 (58%)	109 (84%)
Unknown	48 (10%)	1 (1%)	

**Table 2:** Univariate and multivariate analysis.

	Validation Cohort (GSE73582)				Validation Cohort (GSE25204)			
	Univariate		Multivariate		Univariate		Multivariate	
	HR (95% CI)	*P*	HR (95% CI)	*P*	HR (95% CI)	*P*	HR (95% CI)	*P*
Age (<65 vs. >=65)	1.51 (0.94-2.43)	0.08	1.33 (0.82-2.16)	0.24	0.88 (0.54-1.42)	0.6	0.86 (0.53-1.41)	0.55
Grade (3 vs. 1&2)	1.42 (0.92-2.18)	0.11	1.21 (0.78-1.87)	0.39	0.89 (0.58-1.38)	0.62	0.90 (0.58-1.40)	0.65
Stage (III&IV vs. I&II)	2.57 (1.44-4.61)	0.001	2.57 (1.39-4.73)	0.002				
Debulking (optimal vs. suboptimal)	2.17 (1.37-3.44)	0.001	1.81 (1.12-2.91)	0.01	2.19 (1.45-3.32)	0.0001	1.80 (1.17-2.77)	0.007
miRNA predictor (high vs low risk)	2.04 (1.34-3.10)	0.0008	1.82 (1.17-2.81)	0.007	2.59 (1.72-3.88)	4.46E-06	2.23 (1.46-3.41)	0.0002

## Data Availability

The data used to support the findings of this study are available from the corresponding author upon request.

## Supplementary Materials

Supplementary 1
* Figure S1.* Study design.

Supplementary 2
* Figure S2.* The correlation between VCAN expression and the 4-miRNAs signature expression in the TCGA dataset.

Supplementary 3
* Figure S3.* The correlation between TGF*β* expression and the 4-miRNAs signature expression in the TCGA dataset.

Supplementary 4
* Table S1.* Master regulated analysis results

Supplementary 5
* Table S2.* Patients risk stratification
