# Supplementary material for: Integrative Network Analysis Reveals a MicroRNA-Based Signature for Prognosis Prediction of Epithelial Ovarian Cancer
Source: Biomed Res Int. 2019 Jun 4;2019:1056431. doi: 10.1155/2019/1056431 (PMC6582839; doi:10.1155/2019/1056431)
Supplement: Supplementary 4 — Table S1. Master regulated analysis results [file 1056431.f4.docx]

Table S1. Master regulated analysis results

| **Regulator** | **Regulon Size** | ***P*-value** |
| --- | --- | --- |
| hsa-miR-409-3p | 326 | 0.0053 |
| hsa-miR-200a | 79 | 0.024 |
| hsa-miR-449a | 80 | 0.026 |
| hsa-miR-508-3p | 102 | 0.019 |
| hsa-miR-21* | 230 | 0.062 |
| hsa-miR-214 | 243 | 0.056 |
| hsa-miR-514 | 141 | 0.073 |
| hsa-miR-10b | 85 | 0.31 |
| hsa-miR-136 | 18 | 0.25 |
| hsa-miR-152 | 149 | 0.28 |
| hsa-miR-199a-5p | 111 | 0.25 |
| hsa-miR-214* | 108 | 0.37 |
| hsa-miR-22 | 399 | 0.31 |
| hsa-miR-363 | 22 | 0.34 |
| hsa-miR-376c | 56 | 0.36 |
| hsa-miR-507 | 90 | 0.36 |
| hsa-miR-31 | 25 | 0.4 |
| hsa-let-7b | 173 | 1 |
| hsa-miR-127-3p | 131 | 0.73 |
| hsa-miR-142-3p | 224 | 0.88 |
| hsa-miR-142-5p | 326 | 0.87 |
| hsa-miR-145 | 74 | 0.92 |
| hsa-miR-150 | 87 | 1 |
| hsa-miR-183 | 25 | 1 |
| hsa-miR-192 | 15 | 1 |
| hsa-miR-199b-5p | 87 | 0.51 |
| hsa-miR-20a | 67 | 0.89 |
| hsa-miR-20b | 64 | 0.69 |
| hsa-miR-26b | 21 | 0.69 |
| hsa-miR-376a | 56 | 0.59 |
| hsa-miR-377 | 33 | 0.54 |
| hsa-miR-379 | 100 | 0.81 |
| hsa-miR-381 | 66 | 0.71 |
| hsa-miR-509-3-5p | 19 | 1 |
| hsa-miR-509-3p | 50 | 0.77 |
| hsa-miR-96 | 33 | 0.84 |
