# Supplementary material for: Integrative Network Analysis Reveals a MicroRNA-Based Signature for Prognosis Prediction of Epithelial Ovarian Cancer
Source: Biomed Res Int. 2019 Jun 4;2019:1056431. doi: 10.1155/2019/1056431 (PMC6582839; doi:10.1155/2019/1056431)
Supplement: Supplementary 5 — Table S2. Patients risk stratification [file 1056431.f5.docx]

Table S2. Patients risk stratification

| **ID** | **GEO Accession** | **Cohorts** | **Risk groups** | **Risk score** |
| --- | --- | --- | --- | --- |
| TCGA-04-1331 | TCGA | train | high-risk | 0.613307166 |
| TCGA-04-1332 | TCGA | train | high-risk | 0.456604208 |
| TCGA-04-1336 | TCGA | train | low-risk | 0.253714194 |
| TCGA-04-1337 | TCGA | train | high-risk | 0.895959461 |
| TCGA-04-1338 | TCGA | train | high-risk | 0.472901752 |
| TCGA-04-1341 | TCGA | train | high-risk | 0.498227893 |
| TCGA-04-1342 | TCGA | train | low-risk | 0.311188359 |
| TCGA-04-1343 | TCGA | train | low-risk | 0.368825714 |
| TCGA-04-1346 | TCGA | train | low-risk | 0.323971392 |
| TCGA-04-1347 | TCGA | train | low-risk | 0.27170814 |
| TCGA-04-1348 | TCGA | train | low-risk | 0.324788293 |
| TCGA-04-1349 | TCGA | train | low-risk | 0.418705987 |
| TCGA-04-1350 | TCGA | train | low-risk | 0.373483533 |
| TCGA-04-1356 | TCGA | train | high-risk | 0.740312377 |
| TCGA-04-1357 | TCGA | train | high-risk | 0.479683336 |
| TCGA-04-1361 | TCGA | train | low-risk | 0.323826353 |
| TCGA-04-1362 | TCGA | train | low-risk | 0.23738023 |
| TCGA-04-1364 | TCGA | train | high-risk | 1.006695719 |
| TCGA-04-1365 | TCGA | train | high-risk | 0.462484661 |
| TCGA-04-1367 | TCGA | train | high-risk | 0.698978905 |
| TCGA-04-1514 | TCGA | train | high-risk | 0.630123737 |
| TCGA-04-1517 | TCGA | train | high-risk | 0.617826957 |
| TCGA-04-1519 | TCGA | train | low-risk | 0.404041036 |
| TCGA-04-1525 | TCGA | train | high-risk | 0.812305794 |
| TCGA-04-1530 | TCGA | train | high-risk | 0.600243507 |
| TCGA-04-1542 | TCGA | train | low-risk | 0.104856223 |
| TCGA-04-1638 | TCGA | train | high-risk | 0.519547356 |
| TCGA-04-1646 | TCGA | train | low-risk | 0.389480438 |
| TCGA-04-1648 | TCGA | train | low-risk | 0.121557984 |
| TCGA-04-1649 | TCGA | train | low-risk | 0.133890909 |
| TCGA-04-1651 | TCGA | train | low-risk | 0.068005693 |
| TCGA-04-1652 | TCGA | train | high-risk | 0.611250296 |
| TCGA-04-1654 | TCGA | train | high-risk | 0.512367567 |
| TCGA-04-1655 | TCGA | train | low-risk | 0.148538785 |
| TCGA-09-0364 | TCGA | train | low-risk | 0.361475031 |
| TCGA-09-0366 | TCGA | train | low-risk | 0.397213483 |
| TCGA-09-0367 | TCGA | train | high-risk | 0.538910105 |
| TCGA-09-0369 | TCGA | train | high-risk | 0.703815011 |
| TCGA-09-1662 | TCGA | train | low-risk | 0.426366667 |
| TCGA-09-1666 | TCGA | train | high-risk | 0.465122894 |
| TCGA-09-1669 | TCGA | train | low-risk | 0.299420486 |
| TCGA-09-1670 | TCGA | train | low-risk | 0.191235639 |
| TCGA-09-1673 | TCGA | train | low-risk | 0.206454684 |
| TCGA-09-1674 | TCGA | train | high-risk | 0.675347501 |
| TCGA-09-2045 | TCGA | train | low-risk | 0.290234574 |
| TCGA-09-2048 | TCGA | train | high-risk | 0.916056754 |
| TCGA-09-2050 | TCGA | train | low-risk | 0.315515129 |
| TCGA-09-2051 | TCGA | train | low-risk | 0.094368344 |
| TCGA-09-2054 | TCGA | train | high-risk | 0.608178536 |
| TCGA-10-0926 | TCGA | train | high-risk | 0.678864724 |
| TCGA-10-0927 | TCGA | train | low-risk | 0.330679153 |
| TCGA-10-0928 | TCGA | train | high-risk | 0.658180415 |
| TCGA-10-0930 | TCGA | train | low-risk | 0.378499286 |
| TCGA-10-0931 | TCGA | train | high-risk | 0.510080531 |
| TCGA-10-0933 | TCGA | train | low-risk | 0.176168614 |
| TCGA-10-0934 | TCGA | train | low-risk | 0.122899339 |
| TCGA-10-0935 | TCGA | train | low-risk | 0.357367629 |
| TCGA-10-0936 | TCGA | train | high-risk | 0.804977472 |
| TCGA-10-0937 | TCGA | train | high-risk | 0.485841421 |
| TCGA-10-0938 | TCGA | train | high-risk | 0.776704512 |
| TCGA-13-0714 | TCGA | train | high-risk | 0.553382545 |
| TCGA-13-0717 | TCGA | train | low-risk | 0.291167353 |
| TCGA-13-0720 | TCGA | train | high-risk | 0.765570599 |
| TCGA-13-0723 | TCGA | train | low-risk | 0.359365045 |
| TCGA-13-0724 | TCGA | train | high-risk | 0.47544672 |
| TCGA-13-0725 | TCGA | train | high-risk | 0.472052165 |
| TCGA-13-0726 | TCGA | train | high-risk | 0.515430254 |
| TCGA-13-0727 | TCGA | train | high-risk | 0.558653417 |
| TCGA-13-0730 | TCGA | train | low-risk | 0.279704304 |
| TCGA-13-0751 | TCGA | train | high-risk | 0.638319843 |
| TCGA-13-0755 | TCGA | train | high-risk | 0.848719434 |
| TCGA-13-0757 | TCGA | train | high-risk | 0.5760327 |
| TCGA-13-0761 | TCGA | train | low-risk | 0.424828815 |
| TCGA-13-0762 | TCGA | train | low-risk | 0.374599537 |
| TCGA-13-0764 | TCGA | train | high-risk | 0.69408511 |
| TCGA-13-0765 | TCGA | train | low-risk | 0.349750457 |
| TCGA-13-0766 | TCGA | train | low-risk | 0.425901555 |
| TCGA-13-0768 | TCGA | train | high-risk | 0.481428232 |
| TCGA-13-0791 | TCGA | train | high-risk | 0.567963227 |
| TCGA-13-0792 | TCGA | train | low-risk | 0.414929955 |
| TCGA-13-0793 | TCGA | train | high-risk | 0.578144279 |
| TCGA-13-0794 | TCGA | train | low-risk | 0.281569322 |
| TCGA-13-0795 | TCGA | train | low-risk | 0.39090785 |
| TCGA-13-0797 | TCGA | train | low-risk | 0.297770889 |
| TCGA-13-0799 | TCGA | train | high-risk | 0.664605497 |
| TCGA-13-0800 | TCGA | train | low-risk | 0.417695957 |
| TCGA-13-0801 | TCGA | train | low-risk | 0.436285857 |
| TCGA-13-0802 | TCGA | train | high-risk | 0.573923065 |
| TCGA-13-0803 | TCGA | train | high-risk | 0.527680902 |
| TCGA-13-0804 | TCGA | train | low-risk | 0.312766788 |
| TCGA-13-0805 | TCGA | train | high-risk | 0.46363898 |
| TCGA-13-0883 | TCGA | train | low-risk | 0.338275858 |
| TCGA-13-0885 | TCGA | train | low-risk | 0.323713701 |
| TCGA-13-0886 | TCGA | train | high-risk | 0.451884203 |
| TCGA-13-0887 | TCGA | train | low-risk | 0.308281427 |
| TCGA-13-0888 | TCGA | train | low-risk | 0.250002817 |
| TCGA-13-0889 | TCGA | train | high-risk | 0.512379794 |
| TCGA-13-0890 | TCGA | train | low-risk | 0.417546557 |
| TCGA-13-0891 | TCGA | train | low-risk | 0.259024721 |
| TCGA-13-0897 | TCGA | train | high-risk | 0.67070779 |
| TCGA-13-0899 | TCGA | train | high-risk | 0.724025896 |
| TCGA-13-0903 | TCGA | train | high-risk | 0.502349126 |
| TCGA-13-0904 | TCGA | train | high-risk | 0.47637637 |
| TCGA-13-0906 | TCGA | train | low-risk | 0.162758026 |
| TCGA-13-0910 | TCGA | train | low-risk | 0.438933477 |
| TCGA-13-0911 | TCGA | train | low-risk | 0.425636125 |
| TCGA-13-0912 | TCGA | train | low-risk | 0.406188292 |
| TCGA-13-0913 | TCGA | train | high-risk | 0.571880542 |
| TCGA-13-0916 | TCGA | train | high-risk | 0.472652164 |
| TCGA-13-0919 | TCGA | train | low-risk | 0.421700863 |
| TCGA-13-0920 | TCGA | train | low-risk | 0.444105386 |
| TCGA-13-0921 | TCGA | train | low-risk | 0.219335501 |
| TCGA-13-0923 | TCGA | train | low-risk | 0.414287462 |
| TCGA-13-0924 | TCGA | train | high-risk | 0.531178673 |
| TCGA-13-1403 | TCGA | train | low-risk | 0.383670653 |
| TCGA-13-1404 | TCGA | train | low-risk | 0.444687028 |
| TCGA-13-1405 | TCGA | train | high-risk | 0.533945274 |
| TCGA-13-1407 | TCGA | train | low-risk | 0.332437352 |
| TCGA-13-1408 | TCGA | train | high-risk | 0.611494047 |
| TCGA-13-1409 | TCGA | train | low-risk | 0.296816314 |
| TCGA-13-1410 | TCGA | train | low-risk | 0.339830984 |
| TCGA-13-1411 | TCGA | train | high-risk | 0.498918481 |
| TCGA-13-1412 | TCGA | train | high-risk | 0.611416521 |
| TCGA-13-1477 | TCGA | train | high-risk | 0.640379024 |
| TCGA-13-1481 | TCGA | train | high-risk | 0.614580774 |
| TCGA-13-1482 | TCGA | train | low-risk | 0.388379947 |
| TCGA-13-1483 | TCGA | train | high-risk | 0.591343701 |
| TCGA-13-1484 | TCGA | train | low-risk | 0.408766037 |
| TCGA-13-1485 | TCGA | train | high-risk | 0.504788611 |
| TCGA-13-1487 | TCGA | train | high-risk | 0.770337602 |
| TCGA-13-1488 | TCGA | train | low-risk | 0.352308538 |
| TCGA-13-1489 | TCGA | train | low-risk | 0.318351105 |
| TCGA-13-1491 | TCGA | train | low-risk | 0.330875322 |
| TCGA-13-1492 | TCGA | train | low-risk | 0.238036572 |
| TCGA-13-1494 | TCGA | train | high-risk | 0.579791382 |
| TCGA-13-1495 | TCGA | train | low-risk | 0.401566123 |
| TCGA-13-1496 | TCGA | train | high-risk | 0.620180037 |
| TCGA-13-1497 | TCGA | train | high-risk | 0.699619621 |
| TCGA-13-1498 | TCGA | train | low-risk | 0.300935952 |
| TCGA-13-1499 | TCGA | train | high-risk | 0.579493036 |
| TCGA-13-1500 | TCGA | train | high-risk | 0.469399157 |
| TCGA-13-1501 | TCGA | train | high-risk | 0.560339987 |
| TCGA-13-1504 | TCGA | train | high-risk | 0.700093917 |
| TCGA-13-1505 | TCGA | train | low-risk | 0.398865749 |
| TCGA-13-1506 | TCGA | train | high-risk | 0.663240227 |
| TCGA-13-1507 | TCGA | train | low-risk | 0.298288677 |
| TCGA-13-1509 | TCGA | train | high-risk | 0.540109253 |
| TCGA-13-1510 | TCGA | train | high-risk | 0.490918738 |
| TCGA-13-1511 | TCGA | train | low-risk | 0.392458699 |
| TCGA-13-1512 | TCGA | train | low-risk | 0.367335759 |
| TCGA-13-2060 | TCGA | train | low-risk | 0.297568713 |
| TCGA-20-0987 | TCGA | train | low-risk | 0.163733615 |
| TCGA-20-0990 | TCGA | train | low-risk | 0.422458389 |
| TCGA-20-0991 | TCGA | train | low-risk | 0.330848651 |
| TCGA-20-1682 | TCGA | train | low-risk | 0.378716587 |
| TCGA-20-1683 | TCGA | train | low-risk | 0.236967284 |
| TCGA-20-1684 | TCGA | train | high-risk | 0.630366271 |
| TCGA-20-1685 | TCGA | train | high-risk | 0.502944349 |
| TCGA-20-1686 | TCGA | train | low-risk | 0.425254638 |
| TCGA-20-1687 | TCGA | train | high-risk | 0.514022428 |
| TCGA-23-1022 | TCGA | train | low-risk | 0.430942099 |
| TCGA-23-1024 | TCGA | train | low-risk | 0.083925842 |
| TCGA-23-1027 | TCGA | train | high-risk | 0.833796503 |
| TCGA-23-1028 | TCGA | train | high-risk | 0.552334443 |
| TCGA-23-1030 | TCGA | train | low-risk | 0.341396992 |
| TCGA-23-1031 | TCGA | train | low-risk | 0.339348197 |
| TCGA-23-1032 | TCGA | train | high-risk | 0.462930198 |
| TCGA-23-1107 | TCGA | train | low-risk | 0.449583382 |
| TCGA-23-1109 | TCGA | train | low-risk | 0.446422731 |
| TCGA-23-1110 | TCGA | train | low-risk | 0.449434428 |
| TCGA-23-1111 | TCGA | train | low-risk | 0.291412827 |
| TCGA-23-1113 | TCGA | train | high-risk | 0.549603206 |
| TCGA-23-1116 | TCGA | train | low-risk | 0.414076828 |
| TCGA-23-1117 | TCGA | train | high-risk | 0.490557606 |
| TCGA-23-1118 | TCGA | train | high-risk | 0.57307189 |
| TCGA-23-1119 | TCGA | train | low-risk | 0.215044943 |
| TCGA-23-1120 | TCGA | train | low-risk | 0.296956772 |
| TCGA-23-1121 | TCGA | train | high-risk | 0.653949357 |
| TCGA-23-1122 | TCGA | train | high-risk | 0.542091412 |
| TCGA-23-1123 | TCGA | train | low-risk | 0.417761979 |
| TCGA-23-1124 | TCGA | train | low-risk | 0.326560206 |
| TCGA-23-1809 | TCGA | train | low-risk | 0.118718163 |
| TCGA-23-2072 | TCGA | train | low-risk | 0.299718984 |
| TCGA-23-2077 | TCGA | train | low-risk | 0.155154028 |
| TCGA-23-2078 | TCGA | train | high-risk | 0.525121505 |
| TCGA-23-2079 | TCGA | train | low-risk | 0.260634109 |
| TCGA-23-2081 | TCGA | train | high-risk | 0.644557227 |
| TCGA-23-2084 | TCGA | train | high-risk | 0.772317949 |
| TCGA-24-0966 | TCGA | train | high-risk | 0.666814523 |
| TCGA-24-0968 | TCGA | train | high-risk | 0.597663192 |
| TCGA-24-0975 | TCGA | train | high-risk | 0.656046458 |
| TCGA-24-0979 | TCGA | train | high-risk | 0.569036463 |
| TCGA-24-0980 | TCGA | train | low-risk | 0.304493286 |
| TCGA-24-0982 | TCGA | train | high-risk | 0.502859585 |
| TCGA-24-1103 | TCGA | train | high-risk | 0.659597911 |
| TCGA-24-1104 | TCGA | train | low-risk | 0.39219753 |
| TCGA-24-1105 | TCGA | train | low-risk | 0.326458185 |
| TCGA-24-1413 | TCGA | train | high-risk | 0.688165811 |
| TCGA-24-1416 | TCGA | train | high-risk | 0.622570851 |
| TCGA-24-1417 | TCGA | train | high-risk | 0.564194034 |
| TCGA-24-1418 | TCGA | train | low-risk | 0.276419048 |
| TCGA-24-1419 | TCGA | train | high-risk | 0.586279743 |
| TCGA-24-1422 | TCGA | train | high-risk | 0.665552674 |
| TCGA-24-1423 | TCGA | train | high-risk | 0.50835597 |
| TCGA-24-1424 | TCGA | train | low-risk | 0.355464004 |
| TCGA-24-1425 | TCGA | train | high-risk | 0.774500711 |
| TCGA-24-1426 | TCGA | train | high-risk | 0.452610321 |
| TCGA-24-1427 | TCGA | train | high-risk | 0.514696378 |
| TCGA-24-1428 | TCGA | train | low-risk | 0.279785958 |
| TCGA-24-1430 | TCGA | train | low-risk | 0.357424129 |
| TCGA-24-1431 | TCGA | train | high-risk | 0.48125201 |
| TCGA-24-1434 | TCGA | train | high-risk | 0.630600619 |
| TCGA-24-1435 | TCGA | train | low-risk | 0.326450097 |
| TCGA-24-1436 | TCGA | train | low-risk | 0.445506344 |
| TCGA-24-1463 | TCGA | train | low-risk | 0.440705855 |
| TCGA-24-1464 | TCGA | train | low-risk | 0.334328976 |
| TCGA-24-1466 | TCGA | train | high-risk | 0.502401074 |
| TCGA-24-1467 | TCGA | train | low-risk | 0.345734509 |
| TCGA-24-1469 | TCGA | train | high-risk | 0.720780877 |
| TCGA-24-1470 | TCGA | train | high-risk | 0.47682666 |
| TCGA-24-1471 | TCGA | train | high-risk | 0.814376118 |
| TCGA-24-1474 | TCGA | train | high-risk | 0.679646957 |
| TCGA-24-1544 | TCGA | train | low-risk | 0.304450101 |
| TCGA-24-1545 | TCGA | train | high-risk | 0.580204011 |
| TCGA-24-1546 | TCGA | train | high-risk | 0.468653129 |
| TCGA-24-1548 | TCGA | train | high-risk | 0.455059758 |
| TCGA-24-1549 | TCGA | train | low-risk | 0.358725096 |
| TCGA-24-1550 | TCGA | train | high-risk | 1.036821983 |
| TCGA-24-1551 | TCGA | train | low-risk | 0.432805246 |
| TCGA-24-1552 | TCGA | train | low-risk | 0.40003783 |
| TCGA-24-1553 | TCGA | train | low-risk | 0.346126016 |
| TCGA-24-1555 | TCGA | train | high-risk | 0.646727757 |
| TCGA-24-1556 | TCGA | train | high-risk | 0.778847406 |
| TCGA-24-1557 | TCGA | train | high-risk | 0.567417627 |
| TCGA-24-1558 | TCGA | train | low-risk | 0.327404074 |
| TCGA-24-1560 | TCGA | train | high-risk | 0.513130797 |
| TCGA-24-1562 | TCGA | train | high-risk | 0.756854083 |
| TCGA-24-1563 | TCGA | train | high-risk | 0.609918478 |
| TCGA-24-1564 | TCGA | train | high-risk | 0.529368488 |
| TCGA-24-1565 | TCGA | train | low-risk | 0.437019835 |
| TCGA-24-1567 | TCGA | train | low-risk | 0.291470752 |
| TCGA-24-1603 | TCGA | train | low-risk | 0.437338462 |
| TCGA-24-1604 | TCGA | train | low-risk | 0.39438279 |
| TCGA-24-1614 | TCGA | train | high-risk | 0.799161981 |
| TCGA-24-1616 | TCGA | train | low-risk | 0.115722756 |
| TCGA-24-1842 | TCGA | train | high-risk | 0.54953931 |
| TCGA-24-1843 | TCGA | train | low-risk | 0.373393781 |
| TCGA-24-1844 | TCGA | train | high-risk | 0.592841039 |
| TCGA-24-1845 | TCGA | train | high-risk | 0.628850174 |
| TCGA-24-1846 | TCGA | train | high-risk | 0.464142818 |
| TCGA-24-1847 | TCGA | train | low-risk | 0.362785453 |
| TCGA-24-1849 | TCGA | train | high-risk | 0.552652734 |
| TCGA-24-1850 | TCGA | train | high-risk | 0.488971734 |
| TCGA-24-1920 | TCGA | train | high-risk | 0.606762757 |
| TCGA-24-1923 | TCGA | train | high-risk | 0.453585924 |
| TCGA-24-1924 | TCGA | train | high-risk | 0.450485345 |
| TCGA-24-1928 | TCGA | train | low-risk | 0.330020214 |
| TCGA-24-1930 | TCGA | train | high-risk | 0.535352824 |
| TCGA-24-2019 | TCGA | train | high-risk | 0.515594707 |
| TCGA-24-2020 | TCGA | train | high-risk | 0.866117196 |
| TCGA-24-2023 | TCGA | train | low-risk | 0.021763425 |
| TCGA-24-2024 | TCGA | train | high-risk | 0.577431043 |
| TCGA-24-2026 | TCGA | train | high-risk | 0.678009465 |
| TCGA-24-2027 | TCGA | train | high-risk | 0.632736598 |
| TCGA-24-2029 | TCGA | train | high-risk | 0.459470925 |
| TCGA-24-2030 | TCGA | train | low-risk | 0.403013499 |
| TCGA-24-2033 | TCGA | train | high-risk | 0.585460296 |
| TCGA-24-2035 | TCGA | train | low-risk | 0.399916261 |
| TCGA-24-2036 | TCGA | train | low-risk | 0.087469949 |
| TCGA-24-2038 | TCGA | train | low-risk | 0.427021054 |
| TCGA-24-2254 | TCGA | train | low-risk | 0.130785392 |
| TCGA-24-2260 | TCGA | train | low-risk | 0.397575846 |
| TCGA-24-2261 | TCGA | train | low-risk | 0.335365638 |
| TCGA-24-2262 | TCGA | train | low-risk | 0.196446003 |
| TCGA-24-2267 | TCGA | train | low-risk | 0.237362322 |
| TCGA-24-2271 | TCGA | train | high-risk | 0.615754337 |
| TCGA-24-2280 | TCGA | train | low-risk | 0.367124157 |
| TCGA-24-2281 | TCGA | train | low-risk | 0.256701083 |
| TCGA-24-2288 | TCGA | train | high-risk | 0.552761524 |
| TCGA-24-2289 | TCGA | train | high-risk | 0.652493959 |
| TCGA-24-2290 | TCGA | train | low-risk | 0.396505602 |
| TCGA-24-2293 | TCGA | train | high-risk | 0.72515477 |
| TCGA-24-2297 | TCGA | train | high-risk | 0.509027902 |
| TCGA-24-2298 | TCGA | train | high-risk | 0.472270188 |
| TCGA-25-1312 | TCGA | train | high-risk | 0.531753811 |
| TCGA-25-1313 | TCGA | train | low-risk | 0.387302969 |
| TCGA-25-1314 | TCGA | train | high-risk | 0.468127957 |
| TCGA-25-1315 | TCGA | train | high-risk | 0.560685634 |
| TCGA-25-1316 | TCGA | train | high-risk | 0.490755474 |
| TCGA-25-1317 | TCGA | train | low-risk | 0.413935517 |
| TCGA-25-1318 | TCGA | train | high-risk | 0.629333259 |
| TCGA-25-1319 | TCGA | train | low-risk | 0.375122981 |
| TCGA-25-1320 | TCGA | train | high-risk | 0.603453613 |
| TCGA-25-1321 | TCGA | train | high-risk | 0.632930658 |
| TCGA-25-1322 | TCGA | train | low-risk | 0.344922335 |
| TCGA-25-1323 | TCGA | train | low-risk | 0.397000482 |
| TCGA-25-1324 | TCGA | train | low-risk | 0.387086483 |
| TCGA-25-1325 | TCGA | train | low-risk | 0.362726636 |
| TCGA-25-1326 | TCGA | train | high-risk | 0.472557313 |
| TCGA-25-1328 | TCGA | train | high-risk | 0.535028779 |
| TCGA-25-1329 | TCGA | train | low-risk | 0.405623434 |
| TCGA-25-1623 | TCGA | train | high-risk | 0.453937875 |
| TCGA-25-1625 | TCGA | train | high-risk | 0.531125733 |
| TCGA-25-1626 | TCGA | train | high-risk | 0.62122701 |
| TCGA-25-1627 | TCGA | train | low-risk | 0.36360034 |
| TCGA-25-1628 | TCGA | train | high-risk | 0.603204203 |
| TCGA-25-1630 | TCGA | train | high-risk | 0.611740934 |
| TCGA-25-1631 | TCGA | train | low-risk | 0.248739929 |
| TCGA-25-1632 | TCGA | train | low-risk | 0.428382018 |
| TCGA-25-1633 | TCGA | train | high-risk | 0.807373036 |
| TCGA-25-1634 | TCGA | train | low-risk | 0.282315104 |
| TCGA-25-1635 | TCGA | train | high-risk | 0.479546131 |
| TCGA-25-1870 | TCGA | train | high-risk | 0.452744686 |
| TCGA-25-1871 | TCGA | train | low-risk | 0.392194189 |
| TCGA-25-1877 | TCGA | train | high-risk | 0.535336204 |
| TCGA-25-1878 | TCGA | train | low-risk | 0.415711242 |
| TCGA-25-2042 | TCGA | train | high-risk | 0.790228018 |
| TCGA-25-2391 | TCGA | train | low-risk | 0.17197121 |
| TCGA-25-2392 | TCGA | train | low-risk | 0.355648878 |
| TCGA-25-2393 | TCGA | train | low-risk | 0.218133409 |
| TCGA-25-2396 | TCGA | train | high-risk | 0.626346925 |
| TCGA-25-2397 | TCGA | train | low-risk | 0.236417883 |
| TCGA-25-2398 | TCGA | train | low-risk | 0.410231253 |
| TCGA-25-2399 | TCGA | train | high-risk | 0.711758597 |
| TCGA-25-2400 | TCGA | train | low-risk | 0.122072429 |
| TCGA-25-2401 | TCGA | train | low-risk | 0.388454261 |
| TCGA-25-2404 | TCGA | train | high-risk | 0.559514969 |
| TCGA-25-2408 | TCGA | train | high-risk | 0.815118286 |
| TCGA-25-2409 | TCGA | train | high-risk | 0.584810327 |
| TCGA-29-1688 | TCGA | train | high-risk | 0.736074762 |
| TCGA-29-1690 | TCGA | train | low-risk | 0.244300342 |
| TCGA-29-1691 | TCGA | train | high-risk | 0.602797067 |
| TCGA-29-1693 | TCGA | train | high-risk | 0.455143823 |
| TCGA-29-1694 | TCGA | train | low-risk | 0.424638212 |
| TCGA-29-1695 | TCGA | train | high-risk | 0.644458953 |
| TCGA-29-1696 | TCGA | train | high-risk | 0.526097134 |
| TCGA-29-1697 | TCGA | train | low-risk | 0.193330287 |
| TCGA-29-1698 | TCGA | train | high-risk | 0.574317294 |
| TCGA-29-1699 | TCGA | train | low-risk | 0.289887015 |
| TCGA-29-1701 | TCGA | train | low-risk | 0.400382575 |
| TCGA-29-1702 | TCGA | train | high-risk | 0.726013674 |
| TCGA-29-1703 | TCGA | train | high-risk | 0.572155642 |
| TCGA-29-1705 | TCGA | train | high-risk | 0.62279603 |
| TCGA-29-1707 | TCGA | train | low-risk | 0.405986775 |
| TCGA-29-1710 | TCGA | train | high-risk | 0.45711466 |
| TCGA-29-1711 | TCGA | train | low-risk | 0.227904796 |
| TCGA-29-1761 | TCGA | train | high-risk | 0.507744806 |
| TCGA-29-1762 | TCGA | train | low-risk | 0.261036685 |
| TCGA-29-1763 | TCGA | train | low-risk | 0.21902965 |
| TCGA-29-1764 | TCGA | train | low-risk | 0.395499033 |
| TCGA-29-1766 | TCGA | train | high-risk | 0.455905137 |
| TCGA-29-1768 | TCGA | train | high-risk | 0.579837891 |
| TCGA-29-1769 | TCGA | train | high-risk | 0.542673028 |
| TCGA-29-1770 | TCGA | train | low-risk | 0.395064079 |
| TCGA-29-1771 | TCGA | train | low-risk | 0.369844296 |
| TCGA-29-1774 | TCGA | train | low-risk | 0.236592144 |
| TCGA-29-1775 | TCGA | train | low-risk | 0.146372028 |
| TCGA-29-1776 | TCGA | train | high-risk | 0.775609388 |
| TCGA-29-1777 | TCGA | train | high-risk | 0.485768088 |
| TCGA-29-1778 | TCGA | train | high-risk | 0.516696783 |
| TCGA-29-1781 | TCGA | train | low-risk | 0.166886841 |
| TCGA-29-1783 | TCGA | train | low-risk | 0.426933022 |
| TCGA-29-1784 | TCGA | train | high-risk | 0.65948571 |
| TCGA-29-1785 | TCGA | train | high-risk | 0.499491177 |
| TCGA-29-2414 | TCGA | train | high-risk | 0.608838731 |
| TCGA-29-2425 | TCGA | train | high-risk | 0.454458962 |
| TCGA-29-2427 | TCGA | train | low-risk | 0.283230981 |
| TCGA-29-2428 | TCGA | train | low-risk | 0.241098219 |
| TCGA-30-1714 | TCGA | train | high-risk | 0.617110263 |
| TCGA-30-1718 | TCGA | train | low-risk | 0.371221218 |
| TCGA-30-1853 | TCGA | train | low-risk | 0.170471425 |
| TCGA-30-1855 | TCGA | train | high-risk | 0.462431503 |
| TCGA-30-1856 | TCGA | train | high-risk | 0.47180144 |
| TCGA-30-1857 | TCGA | train | low-risk | 0.357123694 |
| TCGA-30-1859 | TCGA | train | low-risk | 0.372760194 |
| TCGA-30-1860 | TCGA | train | low-risk | 0.330659019 |
| TCGA-30-1861 | TCGA | train | high-risk | 0.576573266 |
| TCGA-30-1862 | TCGA | train | high-risk | 0.597328121 |
| TCGA-30-1866 | TCGA | train | low-risk | 0.263362896 |
| TCGA-30-1867 | TCGA | train | low-risk | 0.413821734 |
| TCGA-30-1880 | TCGA | train | low-risk | 0.295262833 |
| TCGA-30-1887 | TCGA | train | high-risk | 0.626379844 |
| TCGA-30-1891 | TCGA | train | low-risk | 0.418063072 |
| TCGA-30-1892 | TCGA | train | high-risk | 0.724316997 |
| TCGA-31-1944 | TCGA | train | high-risk | 0.668839452 |
| TCGA-31-1946 | TCGA | train | high-risk | 0.638177142 |
| TCGA-31-1950 | TCGA | train | low-risk | 0.105868044 |
| TCGA-31-1951 | TCGA | train | low-risk | 0.340836034 |
| TCGA-31-1953 | TCGA | train | high-risk | 0.534367113 |
| TCGA-31-1956 | TCGA | train | low-risk | 0.36085266 |
| TCGA-31-1959 | TCGA | train | high-risk | 0.462046598 |
| TCGA-36-1568 | TCGA | train | low-risk | 0.293283187 |
| TCGA-36-1569 | TCGA | train | high-risk | 0.472999138 |
| TCGA-36-1570 | TCGA | train | high-risk | 0.734507682 |
| TCGA-36-1571 | TCGA | train | high-risk | 0.920758325 |
| TCGA-36-1574 | TCGA | train | high-risk | 0.868823073 |
| TCGA-36-1575 | TCGA | train | high-risk | 0.521119919 |
| TCGA-36-1576 | TCGA | train | high-risk | 0.676486336 |
| TCGA-36-1577 | TCGA | train | high-risk | 0.520270056 |
| TCGA-36-1578 | TCGA | train | low-risk | 0.304168705 |
| TCGA-36-1580 | TCGA | train | high-risk | 0.505746867 |
| TCGA-36-1581 | TCGA | train | low-risk | 0.412773198 |
| TCGA-57-1582 | TCGA | train | high-risk | 0.673260427 |
| TCGA-57-1583 | TCGA | train | high-risk | 0.468090471 |
| TCGA-57-1584 | TCGA | train | low-risk | 0.190936365 |
| TCGA-57-1585 | TCGA | train | high-risk | 0.556537639 |
| TCGA-57-1586 | TCGA | train | low-risk | 0.366688198 |
| TCGA-57-1993 | TCGA | train | high-risk | 0.460245481 |
| TCGA-57-1994 | TCGA | train | low-risk | 0.312193144 |
| TCGA-59-2348 | TCGA | train | low-risk | 0.267904856 |
| TCGA-59-2350 | TCGA | train | low-risk | 0.213199728 |
| TCGA-59-2351 | TCGA | train | low-risk | 0.2534705 |
| TCGA-59-2352 | TCGA | train | low-risk | 0.100778816 |
| TCGA-59-2354 | TCGA | train | high-risk | 0.795255943 |
| TCGA-59-2355 | TCGA | train | low-risk | 0.428970222 |
| TCGA-59-2363 | TCGA | train | low-risk | 0.30222763 |
| TCGA-61-1721 | TCGA | train | low-risk | 0.336982357 |
| TCGA-61-1724 | TCGA | train | high-risk | 0.557482083 |
| TCGA-61-1725 | TCGA | train | high-risk | 0.67925756 |
| TCGA-61-1728 | TCGA | train | high-risk | 0.482916177 |
| TCGA-61-1733 | TCGA | train | low-risk | 0.41730202 |
| TCGA-61-1737 | TCGA | train | low-risk | 0.384773681 |
| TCGA-61-1738 | TCGA | train | high-risk | 0.527879792 |
| TCGA-61-1740 | TCGA | train | low-risk | 0.277438107 |
| TCGA-61-1741 | TCGA | train | high-risk | 0.570388125 |
| TCGA-61-1743 | TCGA | train | low-risk | 0.227281533 |
| TCGA-61-1895 | TCGA | train | low-risk | 0.408958318 |
| TCGA-61-1899 | TCGA | train | low-risk | 0.140217387 |
| TCGA-61-1900 | TCGA | train | low-risk | 0.428049874 |
| TCGA-61-1901 | TCGA | train | low-risk | 0.288298853 |
| TCGA-61-1904 | TCGA | train | high-risk | 0.472044545 |
| TCGA-61-1906 | TCGA | train | high-risk | 0.752927776 |
| TCGA-61-1907 | TCGA | train | high-risk | 0.552441974 |
| TCGA-61-1910 | TCGA | train | low-risk | 0.261660249 |
| TCGA-61-1913 | TCGA | train | high-risk | 0.594987134 |
| TCGA-61-1914 | TCGA | train | high-risk | 0.47961487 |
| TCGA-61-1915 | TCGA | train | low-risk | 0.339162088 |
| TCGA-61-1917 | TCGA | train | low-risk | 0.395308952 |
| TCGA-61-1918 | TCGA | train | high-risk | 0.557299263 |
| TCGA-61-1919 | TCGA | train | low-risk | 0.400178916 |
| TCGA-61-1995 | TCGA | train | low-risk | 0.31431741 |
| TCGA-61-1998 | TCGA | train | high-risk | 0.676459956 |
| TCGA-61-2000 | TCGA | train | low-risk | 0.238577082 |
| TCGA-61-2002 | TCGA | train | high-risk | 0.620504862 |
| TCGA-61-2003 | TCGA | train | low-risk | 0.376423091 |
| TCGA-61-2008 | TCGA | train | low-risk | 0.434127301 |
| TCGA-61-2009 | TCGA | train | high-risk | 0.571057038 |
| TCGA-61-2012 | TCGA | train | low-risk | 0.31330241 |
| TCGA-61-2016 | TCGA | train | low-risk | 0.144574623 |
| TCGA-61-2088 | TCGA | train | low-risk | 0.303583542 |
| TCGA-61-2092 | TCGA | train | high-risk | 0.530379111 |
| TCGA-61-2094 | TCGA | train | low-risk | 0.204850508 |
| TCGA-61-2095 | TCGA | train | low-risk | 0.083257429 |
| TCGA-61-2097 | TCGA | train | low-risk | 0.156293903 |
| TCGA-61-2098 | TCGA | train | low-risk | 0.418181977 |
| TCGA-61-2101 | TCGA | train | high-risk | 0.509046509 |
| TCGA-61-2102 | TCGA | train | low-risk | 0.268240924 |
| TCGA-61-2104 | TCGA | train | high-risk | 0.720982909 |
| TCGA-61-2109 | TCGA | train | low-risk | 0.238863286 |
| TCGA-61-2110 | TCGA | train | low-risk | 0.316402936 |
| TCGA-61-2111 | TCGA | train | low-risk | 0.326001961 |
| TCGA-61-2113 | TCGA | train | low-risk | 0.437536164 |
| GSM1898488 | GSE73582 | validation 1 | high-risk | 0.854916528 |
| GSM1898489 | GSE73582 | validation 1 | high-risk | 0.660721856 |
| GSM1898490 | GSE73582 | validation 1 | low-risk | 0.59599146 |
| GSM1898491 | GSE73582 | validation 1 | high-risk | 1.126356518 |
| GSM1898492 | GSE73582 | validation 1 | high-risk | 0.685011319 |
| GSM1898493 | GSE73582 | validation 1 | high-risk | 0.737856725 |
| GSM1898494 | GSE73582 | validation 1 | low-risk | 0.651050202 |
| GSM1898495 | GSE73582 | validation 1 | low-risk | 0.17512278 |
| GSM1898496 | GSE73582 | validation 1 | high-risk | 0.971751162 |
| GSM1898497 | GSE73582 | validation 1 | high-risk | 1.125134283 |
| GSM1898498 | GSE73582 | validation 1 | low-risk | 0.388935412 |
| GSM1898499 | GSE73582 | validation 1 | low-risk | 0.270913662 |
| GSM1898500 | GSE73582 | validation 1 | low-risk | 0.495444206 |
| GSM1898501 | GSE73582 | validation 1 | low-risk | 0.301154667 |
| GSM1898502 | GSE73582 | validation 1 | high-risk | 0.775347833 |
| GSM1898503 | GSE73582 | validation 1 | high-risk | 0.785589926 |
| GSM1898504 | GSE73582 | validation 1 | high-risk | 0.866117279 |
| GSM1898505 | GSE73582 | validation 1 | low-risk | 0.524516539 |
| GSM1898506 | GSE73582 | validation 1 | low-risk | 0.560430892 |
| GSM1898507 | GSE73582 | validation 1 | low-risk | 0.174222685 |
| GSM1898508 | GSE73582 | validation 1 | low-risk | 0.51625775 |
| GSM1898509 | GSE73582 | validation 1 | low-risk | 0.374073081 |
| GSM1898510 | GSE73582 | validation 1 | high-risk | 1.012914439 |
| GSM1898511 | GSE73582 | validation 1 | high-risk | 0.873652591 |
| GSM1898512 | GSE73582 | validation 1 | low-risk | 0.492654237 |
| GSM1898513 | GSE73582 | validation 1 | low-risk | 0.377518132 |
| GSM1898514 | GSE73582 | validation 1 | high-risk | 0.713766943 |
| GSM1898515 | GSE73582 | validation 1 | high-risk | 0.850106397 |
| GSM1898516 | GSE73582 | validation 1 | low-risk | 0.477910261 |
| GSM1898517 | GSE73582 | validation 1 | low-risk | 0.644120655 |
| GSM1898518 | GSE73582 | validation 1 | high-risk | 0.818873145 |
| GSM1898519 | GSE73582 | validation 1 | low-risk | 0.215684154 |
| GSM1898520 | GSE73582 | validation 1 | low-risk | 0.626191523 |
| GSM1898521 | GSE73582 | validation 1 | low-risk | 0.573307993 |
| GSM1898522 | GSE73582 | validation 1 | low-risk | 0.430334162 |
| GSM1898523 | GSE73582 | validation 1 | low-risk | 0.464158015 |
| GSM1898524 | GSE73582 | validation 1 | high-risk | 0.942216487 |
| GSM1898525 | GSE73582 | validation 1 | low-risk | 0.481594727 |
| GSM1898526 | GSE73582 | validation 1 | high-risk | 0.833636353 |
| GSM1898527 | GSE73582 | validation 1 | low-risk | 0.579396513 |
| GSM1898528 | GSE73582 | validation 1 | low-risk | 0.252410606 |
| GSM1898529 | GSE73582 | validation 1 | high-risk | 0.820199258 |
| GSM1898530 | GSE73582 | validation 1 | high-risk | 0.656342284 |
| GSM1898531 | GSE73582 | validation 1 | low-risk | 0.081464698 |
| GSM1898532 | GSE73582 | validation 1 | high-risk | 0.934603895 |
| GSM1898533 | GSE73582 | validation 1 | high-risk | 0.791788895 |
| GSM1898534 | GSE73582 | validation 1 | high-risk | 0.773608193 |
| GSM1898535 | GSE73582 | validation 1 | high-risk | 0.857659386 |
| GSM1898536 | GSE73582 | validation 1 | low-risk | 0.25264623 |
| GSM1898537 | GSE73582 | validation 1 | low-risk | 0.609491961 |
| GSM1898538 | GSE73582 | validation 1 | high-risk | 0.890914449 |
| GSM1898539 | GSE73582 | validation 1 | low-risk | 0.115593257 |
| GSM1898540 | GSE73582 | validation 1 | high-risk | 0.830807076 |
| GSM1898541 | GSE73582 | validation 1 | high-risk | 0.910665803 |
| GSM1898542 | GSE73582 | validation 1 | high-risk | 1.144412006 |
| GSM1898543 | GSE73582 | validation 1 | low-risk | 0.61658515 |
| GSM1898544 | GSE73582 | validation 1 | low-risk | 0.400530629 |
| GSM1898545 | GSE73582 | validation 1 | high-risk | 0.76179319 |
| GSM1898546 | GSE73582 | validation 1 | low-risk | 0.605426553 |
| GSM1898547 | GSE73582 | validation 1 | high-risk | 0.940514429 |
| GSM1898548 | GSE73582 | validation 1 | high-risk | 0.854360179 |
| GSM1898549 | GSE73582 | validation 1 | high-risk | 0.88558895 |
| GSM1898550 | GSE73582 | validation 1 | low-risk | 0.312186913 |
| GSM1898551 | GSE73582 | validation 1 | high-risk | 0.892038992 |
| GSM1898552 | GSE73582 | validation 1 | high-risk | 1.086861297 |
| GSM1898553 | GSE73582 | validation 1 | high-risk | 0.877823007 |
| GSM1898554 | GSE73582 | validation 1 | low-risk | 0.466240841 |
| GSM1898555 | GSE73582 | validation 1 | high-risk | 0.821689945 |
| GSM1898556 | GSE73582 | validation 1 | low-risk | 0.324968135 |
| GSM1898557 | GSE73582 | validation 1 | high-risk | 0.831634114 |
| GSM1898558 | GSE73582 | validation 1 | high-risk | 0.66376162 |
| GSM1898559 | GSE73582 | validation 1 | high-risk | 0.929335185 |
| GSM1898560 | GSE73582 | validation 1 | low-risk | 0.366807577 |
| GSM1898561 | GSE73582 | validation 1 | low-risk | 0.280918259 |
| GSM1898562 | GSE73582 | validation 1 | high-risk | 0.827869072 |
| GSM1898563 | GSE73582 | validation 1 | high-risk | 0.839231708 |
| GSM1898564 | GSE73582 | validation 1 | low-risk | 0.442446255 |
| GSM1898565 | GSE73582 | validation 1 | low-risk | 0.478817967 |
| GSM1898566 | GSE73582 | validation 1 | high-risk | 0.900952832 |
| GSM1898567 | GSE73582 | validation 1 | low-risk | 0.360587715 |
| GSM1898568 | GSE73582 | validation 1 | high-risk | 0.652252962 |
| GSM1898569 | GSE73582 | validation 1 | high-risk | 1.009360282 |
| GSM1898570 | GSE73582 | validation 1 | high-risk | 0.985965077 |
| GSM1898571 | GSE73582 | validation 1 | high-risk | 1.008834898 |
| GSM1898572 | GSE73582 | validation 1 | high-risk | 0.751406355 |
| GSM1898573 | GSE73582 | validation 1 | high-risk | 1.160617596 |
| GSM1898574 | GSE73582 | validation 1 | high-risk | 0.801367611 |
| GSM1898575 | GSE73582 | validation 1 | low-risk | 0.549130122 |
| GSM1898576 | GSE73582 | validation 1 | high-risk | 1.072507283 |
| GSM1898577 | GSE73582 | validation 1 | high-risk | 0.741494292 |
| GSM1898578 | GSE73582 | validation 1 | high-risk | 0.950185905 |
| GSM1898579 | GSE73582 | validation 1 | high-risk | 0.833696575 |
| GSM1898580 | GSE73582 | validation 1 | low-risk | 0.534598765 |
| GSM1898581 | GSE73582 | validation 1 | low-risk | 0.427834429 |
| GSM1898582 | GSE73582 | validation 1 | low-risk | 0.590399538 |
| GSM1898583 | GSE73582 | validation 1 | low-risk | 0.47924679 |
| GSM1898584 | GSE73582 | validation 1 | low-risk | 0.615002479 |
| GSM1898585 | GSE73582 | validation 1 | low-risk | 0.342333733 |
| GSM1898586 | GSE73582 | validation 1 | low-risk | 0.391348099 |
| GSM1898587 | GSE73582 | validation 1 | high-risk | 0.696888868 |
| GSM1898588 | GSE73582 | validation 1 | high-risk | 0.904544064 |
| GSM1898589 | GSE73582 | validation 1 | low-risk | 0.581011437 |
| GSM1898590 | GSE73582 | validation 1 | high-risk | 0.824015153 |
| GSM1898591 | GSE73582 | validation 1 | low-risk | 0.373040158 |
| GSM1898592 | GSE73582 | validation 1 | low-risk | 0.45320003 |
| GSM1898593 | GSE73582 | validation 1 | low-risk | 0.502524306 |
| GSM1898594 | GSE73582 | validation 1 | high-risk | 0.877320252 |
| GSM1898595 | GSE73582 | validation 1 | low-risk | 0.48478405 |
| GSM1898596 | GSE73582 | validation 1 | high-risk | 0.686398793 |
| GSM1898597 | GSE73582 | validation 1 | high-risk | 1.026055779 |
| GSM1898598 | GSE73582 | validation 1 | low-risk | 0.563642243 |
| GSM1898599 | GSE73582 | validation 1 | low-risk | 0.541510904 |
| GSM1898600 | GSE73582 | validation 1 | high-risk | 0.746558253 |
| GSM1898601 | GSE73582 | validation 1 | low-risk | 0.513006242 |
| GSM1898602 | GSE73582 | validation 1 | low-risk | 0.470901878 |
| GSM1898603 | GSE73582 | validation 1 | low-risk | 0.376475644 |
| GSM1898604 | GSE73582 | validation 1 | low-risk | 0.432561109 |
| GSM1898605 | GSE73582 | validation 1 | low-risk | 0.402194202 |
| GSM1898606 | GSE73582 | validation 1 | high-risk | 0.950267514 |
| GSM1898607 | GSE73582 | validation 1 | low-risk | 0.373176039 |
| GSM1898608 | GSE73582 | validation 1 | low-risk | 0.114879057 |
| GSM1898609 | GSE73582 | validation 1 | low-risk | 0.494670087 |
| GSM1898610 | GSE73582 | validation 1 | low-risk | 0.234261149 |
| GSM1898611 | GSE73582 | validation 1 | high-risk | 0.821593512 |
| GSM1898612 | GSE73582 | validation 1 | high-risk | 1.019508976 |
| GSM1898613 | GSE73582 | validation 1 | low-risk | 0.630664285 |
| GSM1898614 | GSE73582 | validation 1 | low-risk | 0.429839252 |
| GSM1898615 | GSE73582 | validation 1 | low-risk | 0.210729812 |
| GSM1898616 | GSE73582 | validation 1 | high-risk | 0.891243607 |
| GSM1898617 | GSE73582 | validation 1 | high-risk | 0.786067059 |
| GSM1898618 | GSE73582 | validation 1 | low-risk | 0.443989796 |
| GSM1898619 | GSE73582 | validation 1 | high-risk | 0.903322314 |
| GSM1898620 | GSE73582 | validation 1 | high-risk | 0.927472059 |
| GSM619141 | GSE25204 | validation 2 | high-risk | 0.815619363 |
| GSM619142 | GSE25204 | validation 2 | low-risk | 0.558051512 |
| GSM619143 | GSE25204 | validation 2 | high-risk | 0.643111001 |
| GSM619144 | GSE25204 | validation 2 | low-risk | 0.315479583 |
| GSM619145 | GSE25204 | validation 2 | high-risk | 1.236814299 |
| GSM619146 | GSE25204 | validation 2 | high-risk | 0.999503052 |
| GSM619147 | GSE25204 | validation 2 | high-risk | 0.963702947 |
| GSM619148 | GSE25204 | validation 2 | high-risk | 0.958360709 |
| GSM619149 | GSE25204 | validation 2 | low-risk | 0.334817937 |
| GSM619150 | GSE25204 | validation 2 | low-risk | 0.530959827 |
| GSM619151 | GSE25204 | validation 2 | high-risk | 1.126048001 |
| GSM619152 | GSE25204 | validation 2 | low-risk | 0.356436104 |
| GSM619153 | GSE25204 | validation 2 | high-risk | 0.800367326 |
| GSM619154 | GSE25204 | validation 2 | low-risk | 0.455289319 |
| GSM619155 | GSE25204 | validation 2 | high-risk | 0.810865611 |
| GSM619156 | GSE25204 | validation 2 | high-risk | 0.661250514 |
| GSM619157 | GSE25204 | validation 2 | high-risk | 0.768809096 |
| GSM619158 | GSE25204 | validation 2 | low-risk | 0.036782708 |
| GSM619159 | GSE25204 | validation 2 | low-risk | 0.210266642 |
| GSM619160 | GSE25204 | validation 2 | low-risk | 0.49663106 |
| GSM619161 | GSE25204 | validation 2 | high-risk | 0.834264556 |
| GSM619162 | GSE25204 | validation 2 | high-risk | 1.100450302 |
| GSM619163 | GSE25204 | validation 2 | high-risk | 1.290267486 |
| GSM619164 | GSE25204 | validation 2 | high-risk | 0.815419883 |
| GSM619165 | GSE25204 | validation 2 | low-risk | 0.534178025 |
| GSM619166 | GSE25204 | validation 2 | low-risk | 0.59215523 |
| GSM619167 | GSE25204 | validation 2 | high-risk | 1.242364508 |
| GSM619168 | GSE25204 | validation 2 | high-risk | 0.981592818 |
| GSM619169 | GSE25204 | validation 2 | low-risk | 0.406076406 |
| GSM619170 | GSE25204 | validation 2 | low-risk | 0.307838255 |
| GSM619171 | GSE25204 | validation 2 | high-risk | 0.898526475 |
| GSM619172 | GSE25204 | validation 2 | low-risk | -0.057132386 |
| GSM619173 | GSE25204 | validation 2 | high-risk | 0.775763313 |
| GSM619174 | GSE25204 | validation 2 | high-risk | 0.908814121 |
| GSM619175 | GSE25204 | validation 2 | high-risk | 0.906487171 |
| GSM619176 | GSE25204 | validation 2 | low-risk | 0.417735391 |
| GSM619177 | GSE25204 | validation 2 | high-risk | 0.732460628 |
| GSM619178 | GSE25204 | validation 2 | high-risk | 0.669053884 |
| GSM619179 | GSE25204 | validation 2 | high-risk | 1.357637375 |
| GSM619180 | GSE25204 | validation 2 | low-risk | 0.254571146 |
| GSM619181 | GSE25204 | validation 2 | low-risk | 0.556836289 |
| GSM619182 | GSE25204 | validation 2 | low-risk | 0.393000628 |
| GSM619183 | GSE25204 | validation 2 | low-risk | -0.026494936 |
| GSM619184 | GSE25204 | validation 2 | low-risk | 0.382516466 |
| GSM619185 | GSE25204 | validation 2 | low-risk | 0.444523979 |
| GSM619186 | GSE25204 | validation 2 | low-risk | 0.384516776 |
| GSM619187 | GSE25204 | validation 2 | low-risk | 0.434149062 |
| GSM619188 | GSE25204 | validation 2 | low-risk | 0.500848657 |
| GSM619189 | GSE25204 | validation 2 | high-risk | 0.843253126 |
| GSM619190 | GSE25204 | validation 2 | high-risk | 0.644480203 |
| GSM619191 | GSE25204 | validation 2 | low-risk | 0.29288454 |
| GSM619192 | GSE25204 | validation 2 | low-risk | 0.548525417 |
| GSM619193 | GSE25204 | validation 2 | low-risk | 0.471013662 |
| GSM619194 | GSE25204 | validation 2 | high-risk | 0.926000519 |
| GSM619195 | GSE25204 | validation 2 | low-risk | 0.46696877 |
| GSM619196 | GSE25204 | validation 2 | high-risk | 1.163887068 |
| GSM619197 | GSE25204 | validation 2 | low-risk | 0.263437294 |
| GSM619198 | GSE25204 | validation 2 | high-risk | 0.79340077 |
| GSM619199 | GSE25204 | validation 2 | low-risk | 0.36902768 |
| GSM619200 | GSE25204 | validation 2 | high-risk | 0.948937919 |
| GSM619201 | GSE25204 | validation 2 | high-risk | 1.091057157 |
| GSM619202 | GSE25204 | validation 2 | high-risk | 0.613036372 |
| GSM619203 | GSE25204 | validation 2 | low-risk | 0.354547105 |
| GSM619204 | GSE25204 | validation 2 | low-risk | 0.370094783 |
| GSM619205 | GSE25204 | validation 2 | high-risk | 1.183787928 |
| GSM619206 | GSE25204 | validation 2 | low-risk | 0.241490917 |
| GSM619207 | GSE25204 | validation 2 | high-risk | 1.115292532 |
| GSM619208 | GSE25204 | validation 2 | low-risk | 0.485305052 |
| GSM619209 | GSE25204 | validation 2 | low-risk | 0.525237485 |
| GSM619210 | GSE25204 | validation 2 | high-risk | 0.735565404 |
| GSM619211 | GSE25204 | validation 2 | high-risk | 1.083294548 |
| GSM619212 | GSE25204 | validation 2 | low-risk | 0.164403221 |
| GSM619213 | GSE25204 | validation 2 | low-risk | 0.412715426 |
| GSM619214 | GSE25204 | validation 2 | high-risk | 0.593335035 |
| GSM619215 | GSE25204 | validation 2 | low-risk | 0.380717416 |
| GSM619216 | GSE25204 | validation 2 | high-risk | 1.063987314 |
| GSM619217 | GSE25204 | validation 2 | low-risk | 0.06649904 |
| GSM619218 | GSE25204 | validation 2 | low-risk | 0.582062506 |
| GSM619219 | GSE25204 | validation 2 | high-risk | 0.944417782 |
| GSM619220 | GSE25204 | validation 2 | low-risk | 0.44563882 |
| GSM619221 | GSE25204 | validation 2 | high-risk | 0.826498739 |
| GSM619222 | GSE25204 | validation 2 | high-risk | 0.770986109 |
| GSM619223 | GSE25204 | validation 2 | high-risk | 0.73414587 |
| GSM619224 | GSE25204 | validation 2 | low-risk | 0.274216942 |
| GSM619225 | GSE25204 | validation 2 | low-risk | 0.499147728 |
| GSM1656609 | GSE25204 | validation 2 | high-risk | 0.871884665 |
| GSM1656610 | GSE25204 | validation 2 | high-risk | 0.651294708 |
| GSM1656611 | GSE25204 | validation 2 | low-risk | 0.381182132 |
| GSM1656612 | GSE25204 | validation 2 | low-risk | 0.563652227 |
| GSM1656613 | GSE25204 | validation 2 | high-risk | 0.634766597 |
| GSM1656614 | GSE25204 | validation 2 | low-risk | 0.492093932 |
| GSM1656615 | GSE25204 | validation 2 | low-risk | 0.551395106 |
| GSM1656616 | GSE25204 | validation 2 | high-risk | 1.01863062 |
| GSM1656617 | GSE25204 | validation 2 | high-risk | 0.727227386 |
| GSM1656618 | GSE25204 | validation 2 | low-risk | 0.548843454 |
| GSM1656619 | GSE25204 | validation 2 | high-risk | 0.682980747 |
| GSM1656620 | GSE25204 | validation 2 | low-risk | 0.35702356 |
| GSM1656621 | GSE25204 | validation 2 | low-risk | 0.324122869 |
| GSM1656622 | GSE25204 | validation 2 | low-risk | 0.233987141 |
| GSM1656623 | GSE25204 | validation 2 | low-risk | 0.470293077 |
| GSM1656624 | GSE25204 | validation 2 | high-risk | 0.95930206 |
| GSM1656625 | GSE25204 | validation 2 | high-risk | 1.14910457 |
| GSM1656626 | GSE25204 | validation 2 | high-risk | 0.82942707 |
| GSM1656627 | GSE25204 | validation 2 | low-risk | 0.283243783 |
| GSM1656628 | GSE25204 | validation 2 | high-risk | 0.815270618 |
| GSM1656629 | GSE25204 | validation 2 | high-risk | 0.997132155 |
| GSM1656630 | GSE25204 | validation 2 | high-risk | 0.785729722 |
| GSM1656631 | GSE25204 | validation 2 | high-risk | 0.683268553 |
| GSM1656632 | GSE25204 | validation 2 | high-risk | 0.788988616 |
| GSM1656633 | GSE25204 | validation 2 | low-risk | 0.330439528 |
| GSM1656634 | GSE25204 | validation 2 | low-risk | 0.45531443 |
| GSM1656635 | GSE25204 | validation 2 | low-risk | 0.23266065 |
| GSM1656636 | GSE25204 | validation 2 | low-risk | 0.249117916 |
| GSM1656637 | GSE25204 | validation 2 | low-risk | 0.315124167 |
| GSM1656638 | GSE25204 | validation 2 | high-risk | 1.129551519 |
| GSM1656639 | GSE25204 | validation 2 | high-risk | 0.75982417 |
| GSM1656640 | GSE25204 | validation 2 | low-risk | 0.556415471 |
| GSM1656641 | GSE25204 | validation 2 | low-risk | 0.375144662 |
| GSM1656642 | GSE25204 | validation 2 | low-risk | 0.371490981 |
| GSM1656643 | GSE25204 | validation 2 | high-risk | 0.996420342 |
| GSM1656644 | GSE25204 | validation 2 | low-risk | 0.461162323 |
| GSM1656645 | GSE25204 | validation 2 | low-risk | 0.339286853 |
| GSM1656646 | GSE25204 | validation 2 | low-risk | 0.228398866 |
| GSM1656647 | GSE25204 | validation 2 | high-risk | 0.776913552 |
| GSM1656648 | GSE25204 | validation 2 | low-risk | 0.546070307 |
| GSM1656649 | GSE25204 | validation 2 | high-risk | 0.932085111 |
| GSM1656650 | GSE25204 | validation 2 | high-risk | 1.103093321 |
| GSM1656651 | GSE25204 | validation 2 | high-risk | 0.674059087 |
| GSM1656652 | GSE25204 | validation 2 | high-risk | 1.230525963 |
| GSM1656653 | GSE25204 | validation 2 | high-risk | 0.826667025 |
